# Comparison of methods for spectral alignment and signal modelling of GABA-edited MR spectroscopy data

**DOI:** 10.1016/j.neuroimage.2021.117900

**Published:** 2021-02-27

**Authors:** Reuben Rideaux, Mark Mikkelsen, Richard A.E. Edden

**Affiliations:** a Department of Psychology, Downing Street, University of Cambridge, UK; b Russell H. Morgan Department of Radiology and Radiological Science, The Johns Hopkins University School of Medicine, Baltimore, MD, United States; c F. M. Kirby Research Center for Functional Brain Imaging, Kennedy Krieger Institute, Baltimore, MD, United States

## Abstract

Many methods exist for aligning and quantifying magnetic resonance spectroscopy (MRS) data to measure *in vivo γ*-aminobutyric acid (GABA). Research comparing the performance of these methods is scarce partly due to the lack of ground-truth measurements. The concentration of GABA is approximately two times higher in grey matter than in white matter. Here we use the proportion of grey matter within the MRS voxel as a proxy for ground-truth GABA concentration to compare the performance of four spectral alignment methods (i.e., retrospective frequency and phase drift correction) and six GABA signal modelling methods. We analyse a diverse dataset of 432 MEGA-PRESS scans targeting multiple brain regions and find that alignment to the creatine (Cr) signal produces GABA+ estimates that account for approximately twice as much of the variance in grey matter as the next best performing alignment method. Further, Cr alignment was the most robust, producing the fewest outliers. By contrast, all signal modelling methods, except for the single-Lorentzian model, performed similarly well. Our results suggest that variability in performance is primarily caused by differences in the zero-order phase estimated by each alignment method, rather than frequency, resulting from first-order phase offsets within subspectra. These results provide support for Cr alignment as the optimal method of processing MEGA-PRESS to quantify GABA. However, more broadly, they demonstrate a method of benchmarking quantification of *in vivo* metabolite concentration from other MRS sequences.

## 1. Introduction

*γ*-aminobutyric acid (GABA) is the primary inhibitory neurotransmitter within the human brain. The capacity to estimate the concentration of GABA in vivo using magnetic resonance spectroscopy (MRS) has revealed its diagnostic potential as a biomarker for a variety of neurological and psychiatric disorders, including epilepsy (Bradford, 1995; Olsen and Avoli, 1997), autism (Chao et al., 2010; Markram and Markram, 2010; Rubenstein and Merzenich, 2003; Vattikuti and Chow, 2010), and schizophrenia (Kehrer et al., 2008). The concentration of GABA has also been shown to correlate with individual differences in healthy participants on a range of behavioural tasks (Barron et al., 2016; Edden et al., 2009; Puts et al., 2011; Sumner et al., 2010; Van Loon et al., 2013; Yoon et al., 2010) and neuroimaging signals (Muthukumaraswamy et al., 2012; Stagg et al., 2014; Wang et al., 2017). Further, MRS-detected GABA changes have been associated with learning outcomes (Shibata et al., 2017; Stagg et al., 2011) and processing of sensory stimuli (Lunghi et al., 2015; Rideaux et al., 2019).

In vivo MRS measurements have a relatively low signal-to-noise ratio, requiring a large number of repetitions to be averaged to produce a reliable estimate of metabolite concentration (Mikkelsen et al., 2018). It is typical for there to be variability in the frequency and phase of the acquired transients, and frequency and phase alignment before averaging can improve the signal-to-noise ratio and reduce subject/physiological motion-related variance, especially for difference-edited sequences (Near et al., 2020). Alignment is conceptually straightforward: frequency and phase parameters of individual transients are estimated and then corrected to a common point. However, a number of methods are commonly used to estimate these parameters. Once transients are aligned and averaged, GABA concentration is typically estimated as the area of a model (e.g., a Gaussian) fit to a resonance peak within the difference-edited spectrum. The edited GABA signal is a complex multiplet, the lineshape of which varies slightly depending on sequence and acquisition parameters (Mullins et al., 2014). As such, different models have been applied to quantify the GABA signal (e.g., one or two Gaussians).

There remains no consensus on the optimal way of aligning subspectra or quantifying the GABA signal, as evidenced by the diversity of techniques applied within the literature (Coxon et al., 2018; Rowland et al., 2016; Stagg et al., 2010; Wang et al., 2017, 2014). Benchmarking different methods with *in vivo* data is constrained by the lack of a ground-truth GABA concentration. Benchmarking has been performed using data from phantoms (Jenkins et al., 2019), where the ground truth is known, but this data is qualitatively different from *in vivo* data, altering alignment and modelling strategies. Test-retest reliability has been measured (Brix et al., 2017; Saleh et al., 2016), but does not speak to accuracy. Alignment algorithms have often been developed with reference to their success in reducing subtraction artifacts (Evans et al., 2013; Mikkelsen, Saleh, et al., 2018; Mikkelsen et al., 2020), which is reasonable, but again offers no indication of accuracy. There is some evidence that the concentration of certain pairs of metabolites is related, e.g., GABA and Glx (Steel et al., 2020), so it would be possible to use the strength of the relationship between two related metabolites as an index of accuracy. That is, as the accuracy of estimation is increased, so too will the relationship between measurements. However, as both metabolites are measured from the same spectrum, concentration values are not independent and the relationship between them is subject to multiple confounding factors (e.g., linewidth or poor signal quality could produce spurious correlations). Further, the relationship between metabolites can be regionally variable (Rideaux, 2020, 2021).

Another predictor of MRS-detected GABA is the fractional volume of grey matter within the voxel, as there are differing concentrations of GABA in grey matter and white matter (Harris et al., 2015; Jensen et al., 2005; Mikkelsen et al., 2016; Petroff et al., 1988, 1989), and negligible concentrations in cerebrospinal fluid. In MRS studies, this relationship is often considered a nuisance, and attempts are made to reduce its impact by performing a correction based on the voxel tissue composition (Gasparovic et al., 2006; Harris et al., 2015). The strength of this relationship will be mediated by the accuracy GABA estimates: as estimation accuracy increases, so too will the relationship between GABA and grey matter. Therefore, the relationship between GABA and grey matter provides an index of GABA estimation accuracy. In contrast to neurochemicals measured from the same spectrum, GABA and grey matter measurements are acquired independently, so their relationship is less susceptible to conflation by shared factors.

Using the relationship between GABA and grey matter as an index for GABA estimation accuracy, here we combine two large datasets (totalling 432 difference-edited MEGA-PRESS scans) to compare the performance of different alignment and signal modelling methods. Our primary aim was to assess which alignment and signal modelling methods produces GABA estimates that account for the most variability in the grey matter volume fraction. Our secondary aims were to determine which methods produce the fewest outlier estimates, narrowest signal linewidth, and least (choline) subtraction artifacts.

## 2. Methods

### 2.1. Data collection

Legacy data from previous studies were combined. In these studies, participants underwent MR spectroscopic acquisition targeting visual (*n* = 108; Rideaux, 2020; Rideaux et al., 2019; Rideaux and Welchman, 2018), motor (*n* = 50; Rideaux and Welchman, 2018), dorsolateral prefrontal (*n* = 29), and posterior cingulate cortices (*n* = 29) while at rest (henceforth referred to as Dataset A). These data were combined with the “Big GABA” dataset, comprising MRS data targeting posterior cingulate cortex (n=216; henceforth referred to as Dataset B; Mikkelsen et al., 2017, 2019).

### 2.2. Data acquisition

For Dataset A, MR scanning was conducted on a 3T Siemens Prisma equipped with a 32-channel head coil. Anatomical T1-weighted images were acquired for voxel placement with an MPRAGE sequence: voxel resolution=1 mm^3^ ; TE/TI/TR=3.02/900/2250 ms; scan duration=7.2 min; flip angle=9°; slices=192; FOV=256 mm^2^ ; matrix size=256 × 256; acceleration factor=GRAPPA (2). For detection of GABA+ (GABA + co-edited macromolecules), spectra were acquired using a MEGA-PRESS sequence (Mescher et al., 1998): TE/TR=68/3000 ms; 256 or 400 transients of 2048 data points were acquired, 16 water-unsuppressed transients were additionally acquired; a 14.28 ms Gaussian editing pulse was applied at 1.9 (ON) and 7.5 (OFF) ppm. Water suppression was achieved using variable power with optimized relaxation delays (VAPOR; Tkáč and Gruetter, 2005) and outer volume suppression. Automated shimming followed by manual shimming was conducted to achieve approximately 12 Hz water linewidth.

Dataset B was collected by different sites using similar parameters on GE, Phillips, and Siemens scanners. A detailed description of the data acquisitions for Dataset B can be found in Mikkelsen et al. (2019, 2017). To summarize, MRS targeting posterior cingulate cortex was conducted on 3T Siemens, GE, and Phillips scanners equipped with 8-, 32-, or 64-channel head coils. Spectra were acquired using a MEGA-PRESS sequence: TE=68 ms, TR=2000 ms; 320 transients of either 2048 or 4096 data points were acquired; a 15 ms Gaussian editing pulse was applied at 1.9 (ON) and 7.5 (OFF) ppm. Dataset B was acquired with weaker water-suppression than Dataset A. Henceforth, we will refer to the water suppression used in Dataset A and B as strong and weak water suppression, respectively.

Spectra were acquired from locations targeting visual, motor, dorsolateral prefrontal, and posterior cingulate cortices. See Table 1 for details of voxel location, voxel size, and the number of transients.

**Fig. 1.**
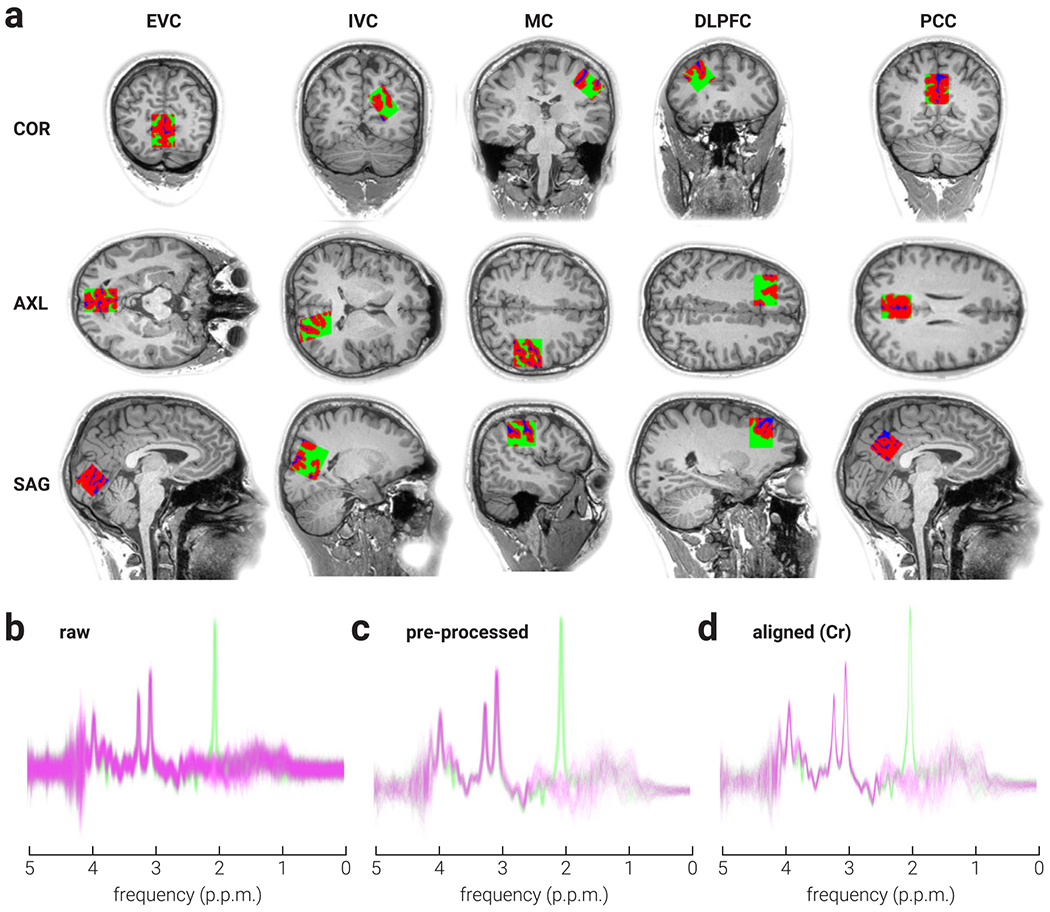
Voxel locations, tissue segmentation, and data processing. a) Coronal (COR), axial (AXL), and sagittal (SAG) views of representative MRS voxel placement and probabilistic partial volume voxel maps for grey matter (red), white matter (green) and cerebrospinal fluid (blue), for early visual (EVC), intermediate visual (IVC), motor (MC), posterior cingulate (PCC), and dorsolateral prefrontal cortices (DLPFC) on a T1-weighted structural image.b-c) Representative example of MRS data from early visual cortex scan (b) before and (c) after pre-processing, and (d) following Cr alignment. Green and magenta lines indicate edit-off and edit-on subspectra, respectively.

The coordinates of the voxel location were used to draw a mask on the anatomical T1-weighted image to calculate the fractional volume of grey matter, white matter, and cerebrospinal fluid within each voxel (Fig. 1). For both Dataset A and B, DICOM files were converted into NIfTI format for tissue segmentation, which was performed using the default settings of the Statistical Parametric Mapping toolbox for MATLAB (SPM12, www.fil.ion.ucl.ac.uk/spm/; Ashburner and Friston, 2005).

### 2.3. Data processing

Spectral pre-processing and quantification were conducted in MATLAB using a combination of Gannet v3.1 (Edden et al., 2014) and in-house scripts. Prior to alignment, subspectra were zero-filled to a spectral resolution of 0.061 Hz/point and 3-Hz exponential line-broadening was applied. For GE and Siemens scans from Dataset B, RF coil combination was also applied.

Four different alignment methods were performed on all data: 1) creatine (Cr) peak alignment, 2) N-acetylaspartate (NAA) peak alignment, 3) spectral registration (SR; Near et al., 2015), and 4) robust spectral registration (rSR; Mikkelsen et al., 2020). We also present results from data with no alignment performed. Alignment methods 1 and 2 were performed in the frequency domain. For Cr and NAA alignment methods, a single-Lorentzian model was fit to either the total creatine (tCr; creatine and phosphocreatine) or total N-acetylaspartate (tNAA; N-acetyl aspartate and N-acetyl aspartyl glutamate) signals to estimate frequency, zero-order phase, area, full-width at half-maximum (FWHM), and baseline and linear offsets. The frequency and phase parameter estimates obtained by modelling the target metabolite signal(s) were then used to align individual subspectra to a common frequency and phase. In the case of NAA alignment, editing-ON spectra that have no NAA signal were assumed to have the same frequency and phase as their corresponding editing-OFF spectra. For subspectra in which a water signal was present (Dataset B), first-order phase was estimated and corrected using the difference in phase between target and water signals. Individual subspectra with frequency, phase, area, or FWHM parameter estimates >3 standard deviations (s.d.) from the mean within a scan were omitted from further analysis. Alignment methods 3 and 4 were performed in the time domain, using a data-driven model to determine the phase and frequency of each transient; for a full description see (Mikkelsen et al., 2020; Near et al., 2015). For methods 3 and 4, individual spectra with parameter estimates of frequency and phase >3 s.d. from the mean within a scan were omitted from further analysis. Where subspectra were omitted, they were done so in (ON and OFF) pairs. An example of raw data before and after pre-processing, and following Cr alignment is shown in Fig. 1b–d.

### 2.4. Signal modelling and metabolite quantification

tCr FWHM was estimated by fitting a double-Lorentzian model to the mean spectrum between 2.5 and 3.5 ppm. tNAA FWHM was estimated by fitting a single-Lorentzian model to the mean OFF spectrum between 1.7 and 2.3 ppm. ON and OFF spectra were subtracted to produce the edited spectrum, from which GABA+ was estimated. Five different models were used to estimate GABA+ signal intensity from the mean edited spectrum between 2.75 and 3.25 ppm: single and double Gaussian, and single, double, and triple Lorentzian. The single-Gaussian model was defined as

(1) $$f\left(x\right)=a\cdot \exp\left(\frac{{\left(x-\mu \right)}^{2}}{2\sigma^{2}}\right)+bx+c$$

where *a* denotes amplitude, *μ* is the centre frequency, *σ* is the s.d., and *b* and *c* are the linear and constant baseline. The double-Gaussian model was the sum of two single Gaussians, including additional *a, μ, σ* free parameters. The single Lorentzian was defined as

(2) $$f\left(x\right)=A\cdot \frac{2\pi^{-1}w}{{\left(x-\mu \right)}^{2}+w^{2}}+bx+c$$

where *A* denotes area, *μ* is the centre frequency, *ω* is the half-width at half-maximum, and *b* and *c* are the linear and constant baseline. Double- and triple-Lorentzian models were the sum of two or three single-Lorentzian models, with additional *A, μ*, *ω* free parameters. We also included model-free estimate of GABA+ by calculating the height of the signal within the GABA+ frequency band. Water signal intensity was estimated by fitting a single-Lorentzian model to the mean unsuppressed spectrum. With the exception of GABA+ height, all GABA+ signal intensities were calculated as the area of the fitted peak(s). GABA+ was then quantified in institutional units (i.u.) using the water signal as an internal concentration reference

(3) GABAiu=AgAw⋅CV⋅1−exp(−TRT1w)1−exp(−TRT1g)⋅exp(−TET2w)exp(−TET2g)⋅ME

where *A* denotes area parameters of GABA+ and water, *C* is the pure water concentration, *V* is the water visibility, *T R* and *T E* are the pulse sequence relaxation and echo times, *T* 1 and *T* 2 are the relaxation times of GABA and water, *M* is the fraction of GABA in the GABA+ signal, and *E* is the editing efficiency of GABA. The following parameters were assumed: pure water concentration, 55000 mmol/L; water visibility, 0.65 (Kreis et al., 1993); T1 and T2 for water, 1.1 and 0.095 (Wansapura, Holland, Dunn, & Ball, 1999); T1 and T2 for GABA, 1.31 and 0.088 (Edden et al., 2012; Puts et al., 2013); fraction of GABA in the GABA+ signal, 0.45; GABA editing efficiency, 0.5 (Near et al., 2011). GABA+ linewidth was calculated as the average FWHM of the three peaks of the triple-Lorentzian model. The phase and FWHM of macromolecule (MM_0.9_) signal were estimated by fitting a complex singlet model to the difference spectrum between 0.5 and 1.5 ppm.

### 2.5. Data screening and statistical analyses

For phase and frequency correlation analyses, estimates >3 s.d. from the mean were omitted prior to calculating the Pearson correlation coefficient. For phase values, the circular correlation coefficient was calculated using the CircStat MATLAB toolbox (Berens, 2009).

For GABA+ analyses, impossible estimates (less than zero) were omitted prior to analyses. The robust correlations MATLAB toolbox (https://sourceforge.net/projects/robustcorrtool; Pernet et al., 2013) was used to calculate Pearson correlations between the proportion of grey matter within the MRS voxel and GABA+ estimates. The boxplot rule was used to omit bivariate outliers, which were excluded from further analyses. The edited spectra from which these bivariate outliers were produced are not included in the plots showing the average edited spectrum. We determined whether correlation coefficients produced using the different alignment methods were significantly higher than the corresponding coefficient produced using no alignment by comparing the *z*-scored coefficients (one-tailed test). For all statistical tests, we used a significance threshold of *p* < .05.

A repeated measures one-way analysis of variance (RM-ANOVA) was used to test for differences between the tCr, tNAA, GABA+, and MM FWHM estimate produced by each alignment method. Unrealistic FWHM estimates (>30 Hz) and estimates >3 s.d. from the mean for each alignment were omitted prior to analysis. Following the RM-ANOVA, paired *t*-tests were conducted between all combinations of alignments. The choline (Cho) subtraction artifact (Evans et al., 2013) was assessed by calculating the standard deviation of signal between 3.16 and 3.285 p.p.m (Mikkelsen et al., 2018). Values >3 s.d. from the mean were omitted prior to analysis.

A power analysis was used to estimate the sample size required to achieve 0.8 statistical power for an independent samples *t*-test where the difference between groups ranged from 5% to 25%. The GABA+ mean and variance used in the calculation were the mean and variance of GABA+ estimates for each signal modelling method, averaged within alignment methods. Values >3 s.d. from the mean were omitted prior to analysis.

## 3. Results

**Fig. 2.**
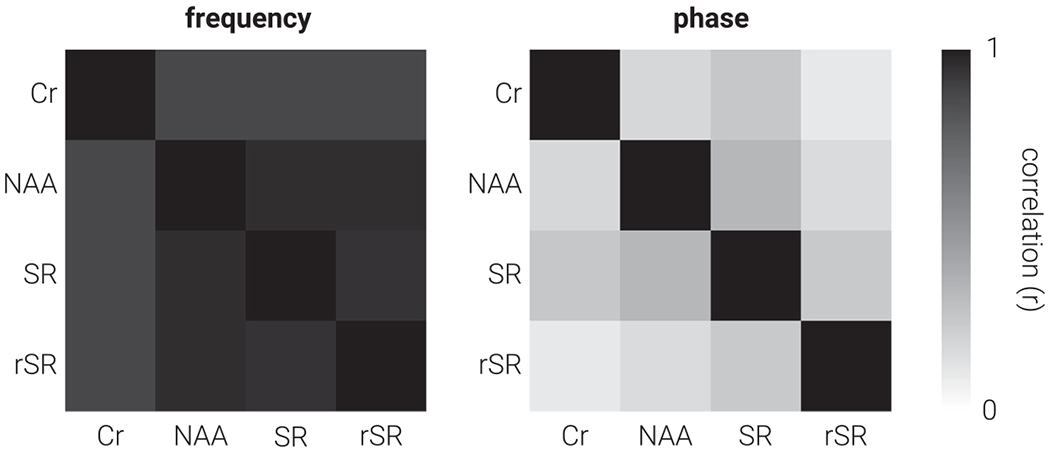
Similarity between alignment methods. Correlation matrices for (left) frequency and (right) phase estimates of subspectra prior to alignment, produced using Creatine (Cr), N-acetylaspartate (NAA), Spectral Registration (SR), and Robust Spectral Registration (rSR) alignment methods.

We computed the similarity between frequency and phase parameters estimated by the different alignment methods (Fig. 2). We found significant positive correlations between frequency and phase for all alignment methods (all *p* < .001). Frequency estimates were more similar than phase estimates; the average correlation coefficient for frequency and phase was .86 and .20, respectively. Although we found significant correlations between frequency and phase estimates produced by the different alignment methods, there was considerable unaccounted variability, especially for phase. This indicates that each method will perform differently, likely producing distinct estimates of neurometabolite concentration.

**Fig. 3.**
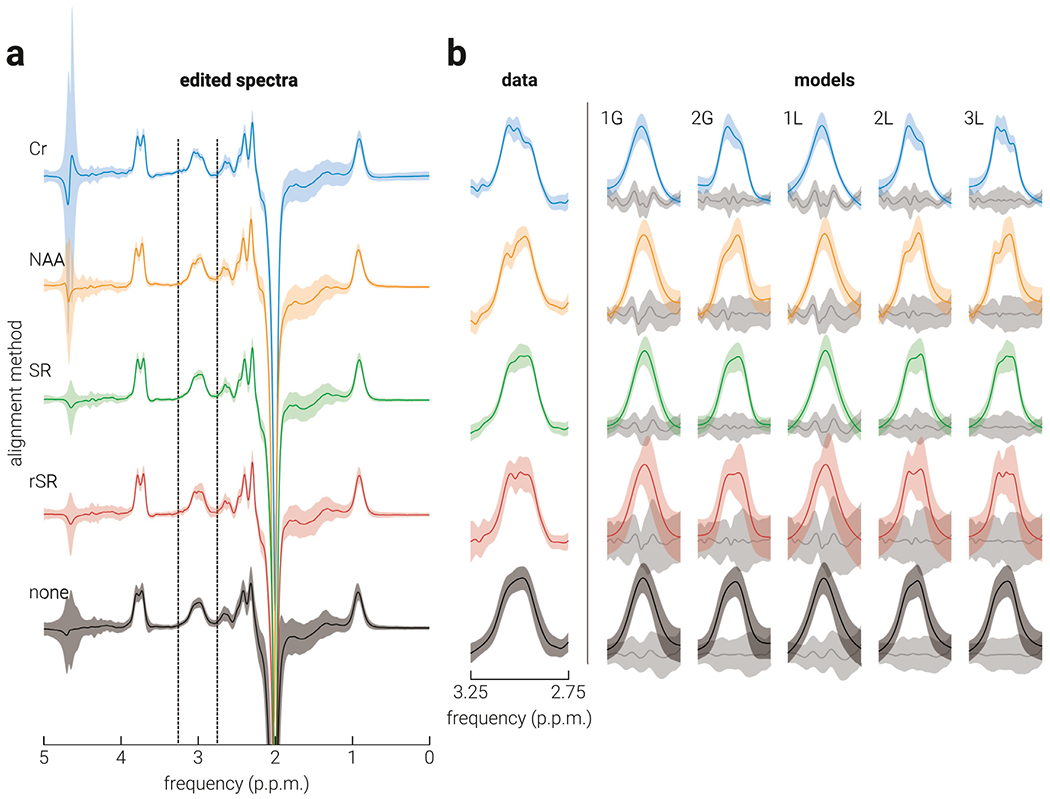
Average edited spectra and GABA+ modelling. a) The average edited spectra produced using Creatine (Cr), N-acetylaspartate (NAA), Spectral Registration (SR), Robust Spectral Registration (rSR), and (none) no alignment methods; vertical dashed lines indicate the frequency band from which GABA+ was estimated. b) The average (left) edited spectra and (right) GABA+ model fit produced using each of the different alignment methods. The amplitude of individual edited spectra and GABA models was normalized to the area of the unsuppressed water peak before being averaged. Grey shaded lines indicate average fit residuals. 1G: single Gaussian; 2G: double Gaussian; 1L: single Lorentzian; 2L: double Lorentzian; 3L: triple Lorentzian. Shaded regions indicate s.d.

Fig. 3a shows the average edited spectra. For all alignment methods, including no alignment (‘none’), the GABA+ signal was visibly present in the average edited spectra. The average GABA+ signal and model fits across all data are shown in Fig. 3b.

**Fig. 4.**
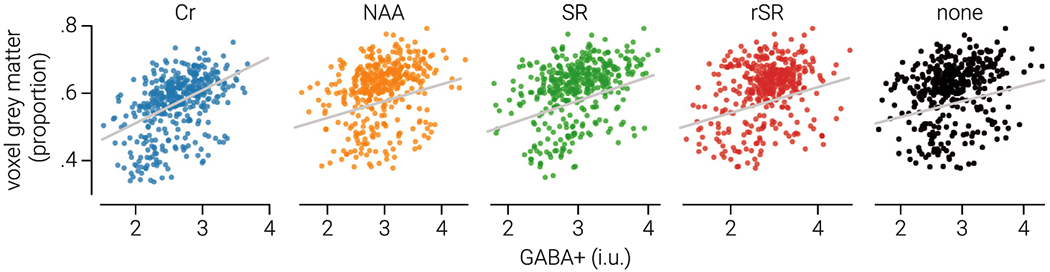
Relationship between GABA+ and grey matter. The proportion of grey matter within the MRS voxel as a function of GABA+ in institutional units (i.u.) as estimated by fitting a single-Gaussian model to spectra produced using Creatine (Cr), N-acetylaspartate (NAA), Spectral Registration (SR), Robust Spectral Registration (rSR), and (none) no alignment methods. Grey lines indicate line-of-best-fit.

Table 2 shows the correlations between grey matter volume fraction (i.e., the faction of grey matter within the voxel) and GABA+ estimated using each combination of alignment and signal modelling method. Fig. 4 shows the relationships for the different alignment methods using a single-Gaussian model. With the exception of NAA alignment, we found that all alignment methods produced at least one set of estimates that were significantly more positively correlated with grey matter than no alignment when combined with a particular GABA model. Overall, Cr alignment performed the best, producing estimates that were significantly more correlated with grey matter than no alignment across four GABA models (1G: *z*_810_=3.25, *p* = 6.0 × 10^−4^; 2G: *z*_790_ = 2.27, *p* = .012; 1L: *z*_824_ = 4.34, *p* = 7.2 × 10^−6^; 3L: *z*_805_ = 2.21, *p* = .013) and significantly more than the next best performing alignment method (SR) across four GABA models (2G: *z*_800_ = 2.86, *p* = .002; 1L: *z*_819_ = 1.86, *p* = .031; 2L: *z*_821_ = 2.67, *p* = .004; 3L: *z*_811_ = 3.38, *p* = 4.0 × 10^−4^). By contrast, there was no clear best performing GABA model; all models performed similarly well, with the exception of the single-Lorentzian model. The triple-Lorentzian model captured the data best, by virtue of having the highest number of free parameters. However, the best performing combination was Cr alignment with a double-Gaussian GABA model, which produced estimates that accounted for 23% of the variance in grey matter. The differences between model performance were not significant, but it is possible that the triple-Lorentzian model did not outperform less complex models because its complexity leaves it more susceptible to the influence of noise and/or artefacts. Note, we found the same pattern of results when we correlated GABA+ with the fraction of grey matter tissue relative to the fraction of white matter tissue and with an index that explicitly assumes GABA is twice a concentrated in grey matter compared to white matter and absent in cerebrospinal fluid, i.e., fGM × 2+fWM.

The average GABA+ peaks produced by the different alignment methods have visible differences in zero-order phase (Fig. 3b, data). To assess the extent to which these differences in phase contributed to the differences in performance between methods, in terms of the correlation between GABA+ and the grey matter volume fraction, we recalculated the correlations between GABA+ and grey matter after regressing the zero-order phase estimates of the MM_0.9_ signal out using linear mixed effects models. We reasoned that if zero-order phase differences contributed to the differences in performance, the correlations between GABA+ and grey matter should be changed significantly as a result of controlling for phase offsets. However, we found that none of the correlations changed significantly as a result of controlling for zero-order phase (all *p* > .05). The Lorentzian models used to estimate GABA+ did not include a phase parameter. Thus, as a further test of the influence of phase offsets on performance, we computed the correlations between GABA+ and grey matter for the Lorentzian models after including an additional phase parameter, which modelled out the phase offset of the GABA+ at the quantification stage. Consistent with the results of the regression analysis, we found the same pattern of results after controlling for zero-order phase at the modelling stage, demonstrating that phase differences in the average difference spectra cannot solely account for the differences observed in performance between the alignment methods.

There are multiple sources of noise that can reduce the quality of MRS data prior to alignment (e.g., participant head movement (Saleh et al., 2020), chemical shift displacement, or dilution associated with changes in blood flow (Ip et al., 2017)), resulting in spurious estimates. Alignment and/or robust model fitting can reduce the proportion of outliers, e.g., by increasing the effective signal-to-noise ratio in the edited spectrum. Here, outliers were excluded separately for each alignment and signal modelling combination, as shown in Table 3. We found that Cr alignment produced the lowest proportion of outliers, while rSR produced the highest.

**Fig. 5.**
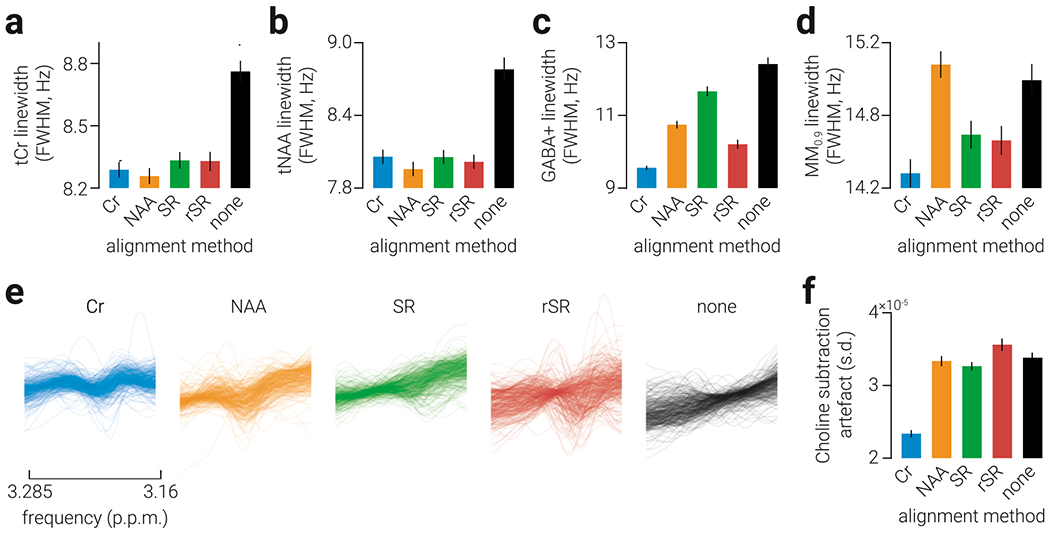
Signal linewidth and Choline subtraction artifact. a-c) Average linewidth of the (a) tCr, (b) tNAA, (c) GABA+, and (d) MM_0.9_ signal produced using different alignment methods. e) Individual edited spectra within the frequency range in which the Cho subtraction artifact occurs (3.16-3.285 ppm). f) The average standard deviation of the edited spectra within the frequency band shown in (e); higher standard deviation is interpreted as increased presence of the Cho subtraction artifact. Error bars indicate s.e.m.

Linewidth is often reported as a measure of spectral quality in MRS studies and can indicate the quality of alignment. We found significant effects of tCr and tNAA FWHM between alignment methods (tCr: *F*_4,1668_ = 162.16, *p* < .001; tNAA: *F*_4,1692_ = 76.69, *p* < .001; Fig. 5a–b). All alignment methods reduced tCr and tNAA signal linewidth compared to no alignment (all *p* < .001) and NAA alignment produced the narrowest linewidth for both signals (all *p* < .001). These results suggest a disconnect between the linewidth of tCr and tNAA signals, and the relationship between GABA+ and grey matter volume fraction. In the difference spectra, we found a significant effect of both GABA+ and MM_0.9_ FWHM (GABA+: *F*_4,1248_ = 68.54, *p* < .001; MM_0.9_: *F*_4,1560_ = 39.70, *p* < .001; Fig. 5c and d). In contrast to the results for tCr and NAA, we found that there was less difference between no alignment and alignment, and Cr alignment produced the narrowest GABA+ and MM_0.9_ linewidth (all *p*<.005). These results are more consistent with the correlations observed between GABA+ estimates and grey matter, and suggest that the linewidth of the difference spectra may be more indicative of GABA+ accuracy.

Modulation of the GABA multiplet between ON and OFF subspectra can introduce an apparent frequency shift in the Cr signal. This shift can impair the alignment and subsequent subtraction of the Cr peak between ON and OFF subspectra, which can introduce a Cho subtraction artifact within the frequency band from which GABA is estimated (Evans et al., 2013). The size of this artifact has previously been used as an index of alignment performance of *in vivo* MRS data (Mikkelsen et al., 2018). We compared the extent of Cho subtraction error between alignment methods (Fig. 5e). We found that Cr alignment produced edited spectra with the smallest Cho subtraction artifact (Fig. 5f). Note that not aligning spectra (‘none’) appears to produce more linear slopes, which is likely due to averaging of misaligned subspectra. These results are in line with the finding that Cr alignment produces estimates that are most predictive of grey matter volume fraction.

**Fig. 6.**
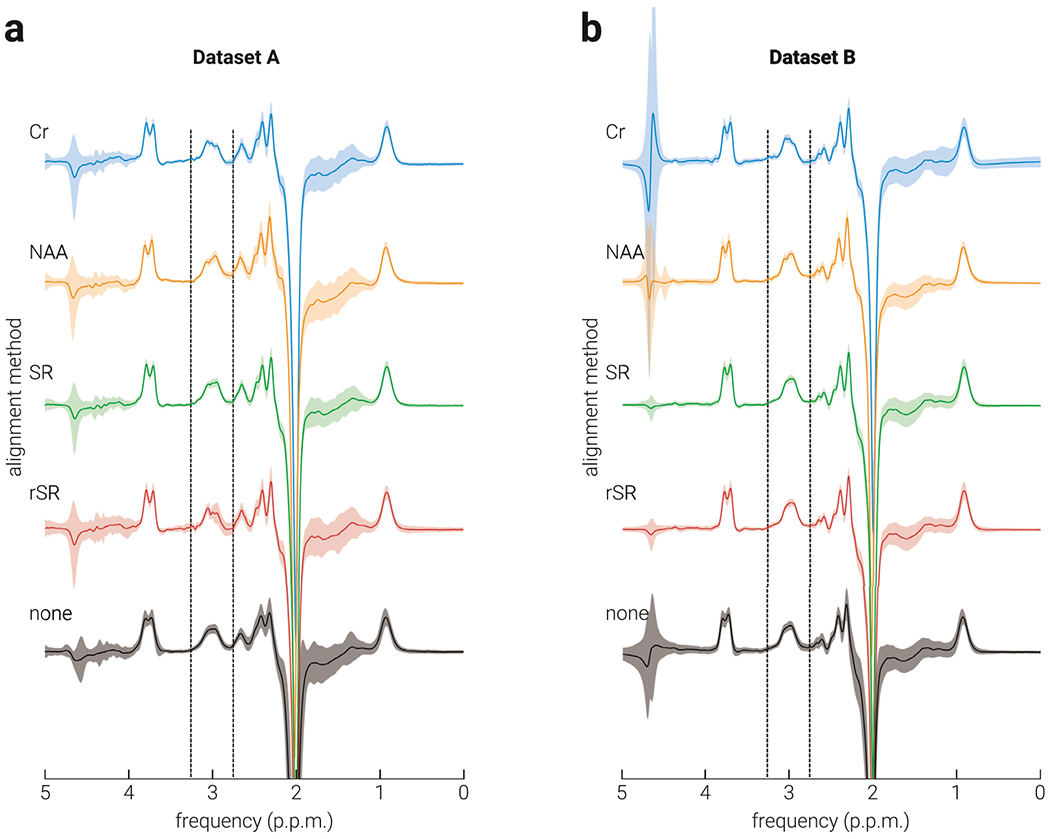
Average edited spectra for dataset A and B. The average edited spectra of dataset a) A and b) B produced using Creatine (Cr), N-acetylaspartate (NAA), Spectral Registration (SR), Robust Spectral Registration (rSR), and (none) no alignment methods; vertical dashed lines indicate the frequency band from which GABA+ was estimated. Shaded regions indicate s.d.

Spectral registration alignment methods, which are performed in the time domain, estimate the frequency and phase of each subspectrum using information across all frequencies. The influence of metabolite signals on the parameter estimates is weighted by their relative amplitude. The strongest signal in the brain is water; thus, if weak water suppression is used to acquire the data, the amplitude of the residual water signal can be orders of magnitude larger than the signals representing other metabolites (e.g., Cr) and will dominate the estimation of frequency and phase. By contrast, the frequency-domain alignment methods (Cr and NAA) only consider information within a relatively narrow frequency band that does not include the residual water signal. This dichotomy can be observed in the average edited spectra of Dataset B (Fig. 6b): there is a minimal presence of water subtraction artifacts at 4.8 ppm in the edited spectra aligned in the time domain, where the water signal is used to estimate frequency and phase. By contrast, there is no such difference in the average edited spectra of Dataset A (Fig. 6a), as these data were collected with stronger water suppression.

Given the reliance of time-domain alignment methods on the water signal, it is possible that the presence of a water signal may moderate the effectiveness of alignment. To test this possibility, we compared the correlation between GABA+ concentration estimates and grey matter volume fraction separately for Dataset A and B, where data were acquired using strong and weak water suppression, respectively (Table 4).

We found larger correlation values between GABA+ estimates and grey matter from Dataset A than Dataset B. This is likely due to the greater variability of grey matter values in Dataset A than Dataset B; Dataset A includes data from voxels targeting five different brain regions, whereas Dataset B includes only voxels targeting posterior cingulate cortex. Diversity among the sequence parameters used to acquire the T1-weighted anatomical image in Dataset B may have also contributed to the relatively weaker correlations by introducing additional variability to the tissue estimates, which is unrelated to GABA+. For both datasets, we found that Cr alignment performed the best, accounting for a maximum of 41% and 18% of the variance in grey matter for Dataset A and B, respectively. However, for Dataset B, the difference in performance between alignment methods was reduced. The only alignment and signal modelling combination that performed significantly better than no alignment was Cr alignment with height estimation. The diagnostic capacity of Dataset B for contrasting alignment methods is likely reduced by the limited variability of grey matter values. However, it may also indicate that alignment methods performed within the time domain, e.g., spectral registration, are as effective at isolating the GABA+ signal as Cr alignment when there is a large residual water signal.

**Fig. 7.**
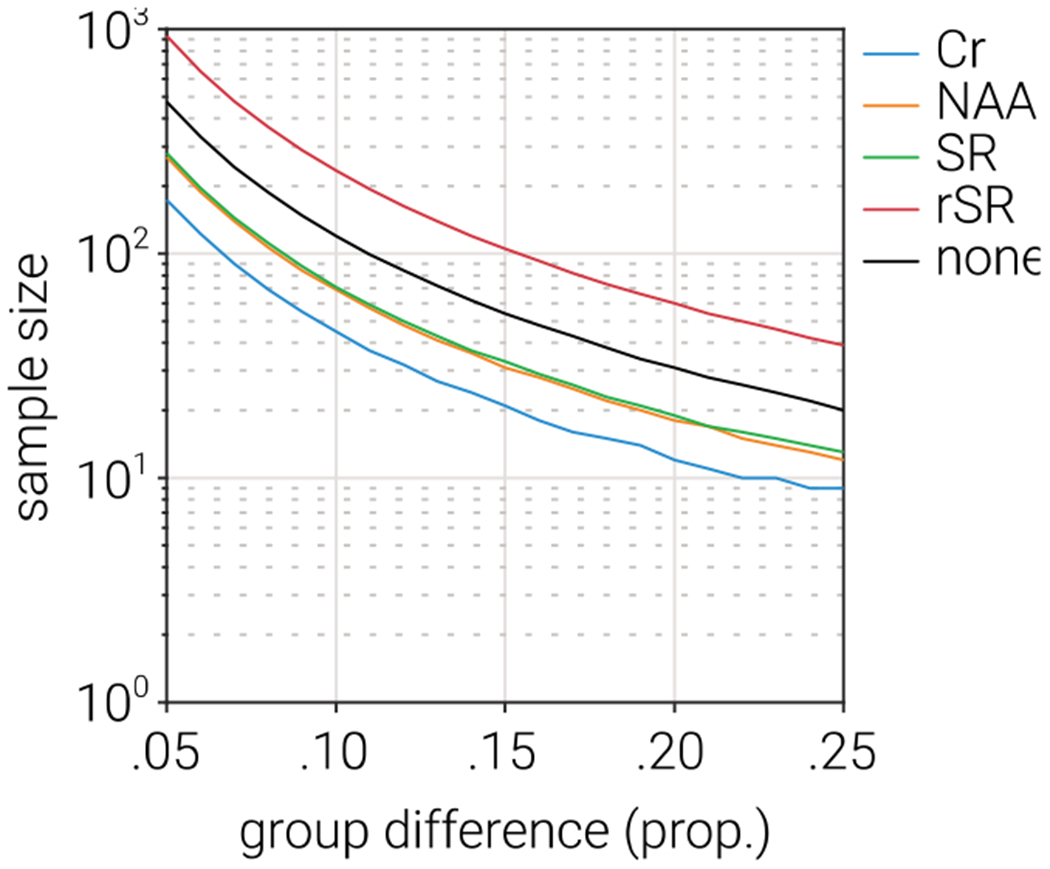
Comparative power analysis of GABA+ estimates produced by different alignment methods. The sample size required to achieve statistical power of 0.8 as a function of group mean difference (independent samples *t*-test), based on the mean and variance of GABA+ estimates produced by each of the alignment methods. The mean and variance were calculated as the average across all signal modelling methods.

To estimate the practical impact of using different alignment methods, we considered the case of comparing two groups in which GABA+ concentration is different, e.g., a patient group compared to a control group. For each alignment method, we calculated the sample size required to achieve statistical power of 0.8 over a range of group differences (Fig. 7). In line with the previous analyses, we found that GABA+ estimates produced by Cr alignment required the fewest subjects to reach sufficient statistical power. For example, with a group mean difference of 15%, statistical power of 0.8 could be reached with 21 subjects when Cr alignment is used, whereas the next best performing alignment method (NAA) required 31 subjects. These results suggest that selecting between different alignment methods can have a powerful influence on the likelihood of detecting differences in GABA+.

## 4. Discussion

Using a dataset of 432 GABA-edited MRS scans, targeting a range of brain regions, we compared four spectral alignment methods and six GABA signal modelling methods, all of which have been used in the literature. We found that alignment to the Cr signal provided GABA+ estimates that were most highly correlated with grey matter, accounting for 23% of the variance. We further found that Cr alignment produces the fewest outliers and the least Cho subtraction error. By contrast, we find that all the signal modelling methods performed similarly well, with the exception of modelling the GABA signal with a single Lorentzian, which performed relatively poorly.

Two frequency-domain (Cr and NAA) and two time-domain (spectral registration and robust spectral registration) alignment methods were compared. Each approach has theoretical benefits and drawbacks; for instance, the *in vivo* concentration of NAA in the brain is generally higher than Cr, providing a signal with a higher signal-to-noise ratio from which to estimate frequency and phase parameters. However, in difference-edited spectra, the NAA signal is suppressed in the ON subspectra, which means that correction parameters can only be estimated for half of the total subspectra. While frequency-domain alignment methods use only a relatively small proportion of the MR spectrum to estimate frequency and phase, time-domain alignment methods use the entire MR spectrum and are therefore more robust to noise, but susceptible to changes in lipid signals or the degree of water suppression.

We found that GABA+ estimates produced by Cr alignment accounted for almost twice as much of the variance in grey matter (23%) as the next best alignment (NAA, 14%). One possible explanation for this is that the frequency of the Cr signal (3.02 ppm) is closest to the GABA signal (3.0 ppm). If there are first-order phase differences across subspectra, which are left uncorrected, these will have an increasing influence on the subspectra as a function of the distance from the frequency at which the zero-order phase is estimated and corrected. The frequency of the NAA signal is further from the GABA signal than Cr, thus first-order phase differences will produce a more misaligned phase at the GABA frequency. This may also explain why Cr alignment produced the least Cho subtraction error. We performed first-order phase correction for Cr and NAA alignment methods; however, accurate estimation of the first-order phase requires at least two signals that are sufficiently separated in frequency. Thus, we could only perform this correction in Dataset B, where a reference water signal was present, and only for half of the NAA subspectra within each scan. Further, even if first-order phase correction could be performed on all subspectra, the accuracy with which this parameter is estimated is imperfect and will introduce an additional source of noise that can instead be minimised by correcting the zero-order phase at a frequency close to the target metabolite (e.g., GABA). The variability in phase estimated by the alignment methods is consistent with the presence of first-order phase offsets within the subspectra and implicates phase, rather than frequency, correction as the primary factor driving differences in performance between alignment methods. Currently, neither of the time-domain-based alignment methods include first-order phase correction. It is possible that the inclusion of this additional correction may improve the performance of these methods.

With the exception of the single-Lorentzian model, which performed relatively poorly, the average performance of the signal models we compared was similar (note, the double-Gaussian model performed the best). This indicates that these signal modelling methods provide highly correlated estimates of the same data (Mullins et al., 2014). Further, this implies that the selection of the alignment method is more important than that of the GABA signal modelling method.

Half of all the scans used in the current study (Dataset A) were collected using strong water suppression, while the other half (Dataset B) were collected using weaker water suppression. Where strong water suppression was used, almost no residual water signal was present in the spectrum. By contrast, where weaker water suppression is used, a residual water signal is present that is orders of magnitude larger than the other metabolite signals. For the frequency-domain alignment methods, the presence/absence of a water signal should have minimal influence as frequency and phase parameters are estimated within a frequency band that excludes the water signal. By contrast, for time-domain alignment methods, the water signal has a considerable influence on the estimation of correction parameters. We assessed whether the presence/absence of a water signal changed the relative performance of the alignment methods by analysing Dataset A and B separately. We found that the difference in performance between Cr alignment and time-domain alignments was larger for scans with strong water suppression than for scans with weak water suppression. This suggests that time-domain alignments may perform as well as Cr alignment when a water signal is present in the spectrum. However, grey matter volume fraction was less variable between the scans with weak water suppression, which also likely reduced the differences between the correlations with GABA+. Further, although the difference between correlations is reduced, it is reassuring to find the same pattern of results between Datasets A and B, which were collected by different research groups using different scanning protocols.

Previous work has assessed the performance of alignment and signal modelling methods by using phantom data (Jenkins et al., 2019), or measuring test-retest reliability (Brix et al., 2017; Saleh et al., 2016) and subtraction artifacts (Evans et al., 2013; Mikkelsen et al., 2018, 2020). However, it is unclear how well results from the analysis of phantom data translate to data collected *in vivo*, and reliability and the absence of artifacts do not necessarily indicate accuracy. The benchmarking method applied in the current study sought to overcome these limitations by using the proportion of grey matter as a proxy for *in vivo* ground truth GABA concentration. However, there are limitations associated with this method. First, factors other than the fraction of grey matter within the MRS voxel influence the concentration GABA, which limits its accuracy as a proxy. For example, we pooled data from different cortical locations, which provided better diagnostic capacity by increasing the variability of grey matter volume fractions. However, GABA_A_ receptors are not uniformly expressed within the neocortex across the brain (Lee et al., 2014), so the correlation between GABA and grey matter likely varies as a function of brain region. Controlling for differences in cortical location prior to correlating GABA+ and grey matter is an option, but this would reduce the diagnostic capacity of the test. More work is needed to assess the relationship between grey matter and GABA as a function of brain region. Second, although grey matter and GABA estimates are collected in different scans, the degree of participant movement during scans is likely to be related and it is possible that this may influence both estimates in the same way. For example, during the anatomical scan, excessive participant motion may reduce the estimated grey matter by blurring the boundaries between tissue compartments, and, during the MRS scan, it may reduce the GABA signal by introducing additional noise. Finally, the accuracy of GABA+ is a relatively downstream metric from which to judge the performance of alignment. This method relies on the assumption that there is a relationship between GABA+ and grey matter volume fraction. Although we have strong evidence supporting this relationship, more common upstream metrics such as linewidth are not dependent on this assumption. These limitations underscore the importance of considering multiple lines of evidence when comparing performance and it is encouraging that we found Cr alignment also produced the fewest outliers, the narrowest GABA+ and MM_0.9_ linewidth, and smallest Cho subtraction error. Here we assessed the performance of different alignment and signal modelling methods for GABA-edited data. However, future work could apply this benchmarking technique to data acquired using other MRS sequences, e.g., non-edited sequences, or to optimise alignment and quantification methods. Closed source software packages (e.g., LCModel, Tarquin, JMRUI) remain a popular means of analysing MRS data, so future work could also use the benchmarking technique developed here to compare their performance.

There are currently multiple different methods of analysing MRS data to measure GABA concentration, all of which produce different estimates. Here we use the proportion of grey matter within the MRS voxel as a proxy for *in vivo* GABA ground truth to benchmark the optimal method. We find that alignment to the Cr signal produces GABA+ estimates that account for twice as much of the variance in grey matter as the next best performing alignment. These results provide support for Cr alignment as the optimal method of processing MEGA-PRESS data for the purpose of quantifying GABA, but more broadly, they demonstrate a means of benchmarking quantification of other metabolites and analyses of MRS data from different sequences.

## Figures and Tables

**Table 1:** Details of Dataset A scans.

Voxel location (cortex)	Voxel size (cm^3^)	# of transients	# of scans	Voxel placement description
early visual	3 × 3 × 2 ( *n* = 40) 2.5 × 2.5 × 2.5 (*n* = 29)	256	69	medially in the occipital lobe; the lower face aligned with the cerebellar tentorium
intermediate visual	3 × 3 × 2	256	39	right hemisphere, adjacent to the median line, and rotated in the sagittal and axial planes so as to align with the posterior surface of the brain
Motor	3 × 3 × 2	256 (*n* = 19) 400 (*n* = 31)	50	right hemisphere, centred on the ‘hand knob’ area of the precentral gyrus and aligned to the upper surface of the brain in the sagittal and coronal planes
posterior cingulate	2.5 × 2.5 × 2.5 3 × 3 × 3	256 320	29 216	centred on the medial parietal lobe and rotated in the sagittal plane to align with a line connecting the genu and splenium of the corpus callosum
dorsolateral prefrontal	2.5 × 2.5 × 2.5	256	29	left hemisphere, above the superior margin of the lateral ventricles

**Table 2:** Correlation coefficients between GABA+ and grey matter voxel tissue fraction.

Alignment method	GABA signal modelling method						
	1G	2G	1L	2L	3L	Height	Average
Cr	.45***	.48*	.38***	.42	.46*	.33	.42
NAA	.28	.34	.05	.33	.37	.43	.30
SR	.36*	.31	.27**	.25	.25	.35	.30
rSR	.29	.34	.30**	.33	.35	.15	.29
none	.25	.34	.10	.32	.32	.41	.29
average	.33	.36	.22	.33	.35	.34	.32

Note: 1G: single Gaussian; 2G: double Gaussian; 1L: single Lorentzian; 2L: double Lorentzian; 3L: triple Lorentzian. Single, double, and triple asterisks indicate correlation coefficients that are significantly higher (*p*<[.05, .01, and .001], respectively) than the corresponding coefficient for no alignment (‘none’).

**Table 3:** Percentage of bivariate outliers.

Alignment method	GABA signal modelling method						
	1G	2G	1L	2L	3L	Height	Average
Cr	4.6	5.8	4.4	3.7	4.6	5.3	4.8
NAA	6.5	8.3	3.9	7.9	5.8	5.6	6.3
SR	7.2	8.6	5.6	5.8	7.2	6.5	6.8
rSR	6.0	10.9	6.3	6.0	8.3	13.0	8.4
none	7.4	10.9	4.4	7.9	8.6	6.9	7.7
average	6.3	8.9	4.9	6.3	6.9	7.5	6.8

Note: 1G: single Gaussian; 2G: double Gaussian; 1L: single Lorentzian; 2L: double Lorentzian; 3L: triple Lorentzian. Total sample size = 432.

**Table 4:** Correlation coefficients between GABA+ and grey matter volume fraction with strong and weak water suppression.

Alignment method	GABA signal modelling method						
	1G	2G	1L	2L	3L	Height	Average
Cr	.61**|.30	.55|.34	.64***|.22	.52|.33	.54|.34	.26|.43**	.52|.33
NAA	.38|.29	.41|.23	.15|.23	.41|.21	.47|.27	.57|.24	.40|.24
SR	.50|.27	.35|.23	.47**|.25	.32|.25	.29|.25	.37|.33	.38|.27
rSR	.28|.22	.31|.16	.27|.19	.26|.23	.26|.25	.25|.33	.27|.23
none	.38|.28	.45|.22	.26|.27	.44|.25	.47|.23	.50|.21	.42|.24
average	.43|.27	.41|.23	.36|.23	.39|.26	.40|.27	.39|.31	.40|.26

Note: 1G: single Gaussian; 2G: double Gaussian; 1L: single Lorentzian; 2L: double Lorentzian; 3L: triple Lorentzian. Correlation coefficients corresponding to data from the two datasets are shown in the format (Dataset A)|(Dataset B). Single, double, and triple asterisks indicate correlation coefficients that are significantly higher (*p*<[05, .01, and 0.001] respectively) than the corresponding coefficient for no alignment (‘none’).
